# Anti-Proliferative Effects of Compound A and Its Effect in Combination with Cisplatin in Cholangiocarcinoma Cells

**DOI:** 10.31557/APJCP.2020.21.9.2673

**Published:** 2020-09

**Authors:** Mutita Junking, Thidarath Rattanaburee, Aussara Panya, Irina Budunova, Guy Haegeman, Pa-Thai Yenchitsomanus

**Affiliations:** 1 *Division of Molecular Medicine, Research Department, Faculty of Medicine Siriraj Hospital, Mahidol University, Bangkok, Thailand. *; 2 *Siriraj Center of Research Excellence for Cancer Immunotherapy (SiCORE-CIT), Faculty of Medicine Siriraj Hospital, Mahidol University, Bangkok, Thailand. *; 3 *Graduate Program in Immunology, Department of Immunology, Faculty of Medicine Siriraj Hospital, Mahidol University, Bangkok, Thailand. *; 4 *Department of Biology, Faculty of Science, Chiang Mai University, Chiang Mai, Thailand. *; 5 *Center of Excellence in Bioresources for Agriculture, Industry and Medicine, Faculty of Science, Chiang Mai University, Chiang Mai, Thailand. *; 6 *Department of Dermatology, Feinberg School of Medicine, Northwestern University, Chicago, Illinois, USA. *; 7 *Laboratory of Eukaryotic Gene Expression and Signal Transduction (LEGEST), Department of Physiology, Faculty of Sciences, Ghent University, Ghent, Belgium. *

**Keywords:** Cholangiocarcinoma, bile duct cancer, glucocorticoid, compound A, interleukin-6

## Abstract

**Background:**

Cholangiocarcinoma (CCA) is a fatal cancer with high resistance to anticancer drugs. The development of new drugs or compounds to be used alone or in combination with currently available chemotherapeutic agents to improve the treatment of CCA is needed. Compound A (CpdA), which is a small plant-derived glucocorticoid receptor modulator, strongly inhibited the growth and survival of several cancers. However, the effect of CpdA on cholangiocarcinoma has not been elucidated. The aim of this study was to investigate the effect of CpdA on CCA.

**Methods:**

Cytotoxicity of CpdA was tested in primary cells including peripheral blood mononuclear cells (PBMCs), fibroblasts, and human umbilical vein endothelial cells (HUVECs), as well as on CCA cell lines (KKU-100, KKU-055, and KKU-213) was examined. Cell cycle distribution and IL-6 expression was assessed by flow cytometry and real-time polymerase chain reaction, respectively. The effect of combination CpdA and cisplatin was evaluated by cell viability assay.

**Results:**

CpdA significantly inhibited cell cycle at G1 phase in CCA cell lines, and reduced IL-6 mRNA expression. However, combination CpdA and cisplatin did not enhance the inhibitory effect. TGFβR-II expression was increased in CCA cells after the combination treatment.

**Conclusions:**

These results indicate the potential of CpdA for CCA treatment. However, combination treatment with CpdA and cisplatin increased CCA cell survival. The molecular mechanism is likely attributable to promotes cell survival via the TGFβR-II signaling pathway. The combination of CpdA with other anticancer drugs for CCA treatment should be further examined.

## Introduction

Cholangiocarcinoma (CCA) is an aggressive and lethal cancer that originates from biliary epithelia within the biliary tract (Kambakamba and DeOliveira, 2014). This cancer is rare worldwide, but its incidence is increasing and it is the most common liver cancer in Northeastern Thailand (Sripa and Pairojkul, 2008). Infection with the liver fluke Opisthorchis viverrini is the most common risk factor for CCA in Thailand (Sithithaworn et al., 2014). CCA is normally difficult to diagnose until the disease becomes advanced. Surgery is the only curative treatment for patients with CCA. However, CCA is well known to recur after surgery, and the mortality rate is high (Rosen et al., 2008). The studies that investigated the use of chemotherapy to treat CCA were small, the results they reported were conflicting, and the patient outcomes were poor (Nakajima et al., 2003; Yoon et al., 2011; Lin et al., 2012). The aggressive and unresponsive nature of this disease highlights the need to urgently identify new drugs or compounds to be used alone or in combination with currently available chemotherapeutic agents to improve the treatment of CCA.

The carcinogenesis of CCA is a multistep cellular process (Fava et al., 2007; Fava and Lorenzini, 2012). CCA cells express altered molecular mechanisms that enhance cell proliferation, decrease apoptosis, and increase the capacity of tissue invasion. Inflammatory cytokines, such as IL-6, play an important role in the pathogenesis and growth of CCA by activating several pathways involved in survival signaling, and they also contribute to chemoresistance (Okada et al., 1994; Park et al., 1999a; Park et al., 1999b). Glucocorticoids (GC) are potent anti-inflammatory agents that are used in chronic inflammatory diseases and in many cancers. Dexamethasone, which is a synthetic GC, was used to treat CCA in a phase II clinical trial, but various metabolic side effects were observed (Pazdur et al., 1999). A small plant-derived glucocorticoid receptor (GR) modulator, 2-(4-acetoxyphenyl)-2-chloro-N-methylethylammonium-chloride, also called compound A (CpdA), was recently shown to prevent GR dimerization, preserve glucocorticoid anti-inflammatory activity, and have fewer side effects compared with glucocorticoids. CpdA is a plant-derived phenyl aziridine precursor that is extracted from Salsola tuberculatiformis Botschantzev, a Namibian shrub (Louw et al., 1997; Louw and Swart, 1999; De Bosscher et al., 2005). CpdA also demonstrates anti-inflammatory activity (Dewint et al., 2008; Gossye et al., 2009; Rauch et al., 2011), and it strongly inhibits the growth and survival of prostate cancer and multiple myeloma cells by reducing the release of inflammatory cytokines via inhibition of NF-kB and AP-1 transcriptional factors (Yemelyanov et al., 2012; Lesovaya et al., 2013; Lesovaya et al., 2015). The fact that CpdA strongly interacts with GR, and that it instigates inhibition of cancer cell progression suggests that CpdA may have inhibitory effects on CCA.

In this study, we set forth to evaluate the anticancer effect of CpdA on CCA cell lines. The cytotoxicity of CpdA on primary cells, including peripheral blood mononuclear cells (PBMCs), fibroblasts, and human umbilical vein endothelial cells (HUVECs), as well as on CCA cell lines (KKU-100, KKU-055, and KKU-213) was examined. The effect of CpdA at sublethal dose on cell cycle distribution and IL-6 expression was determined by real-time polymerase chain reaction (PCR) and flow cytometry, respectively. The anticancer activities of CpdA on cell survival were investigated using CpdA alone and in combination cisplatin, which is a conventional anticancer agent. The possible mechanism of CpdA-induced cisplatin-resistance was also studied.

## Materials and Methods

*CCA cell lines and skin fibroblast cells*

Human CCA cell lines KKU-100 (JCRB1568), KKU-055 (JCRB1551), and KKU-213 (JCRB1557) were obtained from the Japanese Collection of Research Bioresources Cell Bank (JCRB), National Institute of Biomedical Innovation, Osaka Japan. These cell lines were opisthorchiasis-associated CCA that were established and characterized at the Liver Fluke and Cholangiocarcinoma Research Center, Khon Kaen University, Khon Kaen, Thailand. These cell lines were deposited at the JCRB for long-term storage and for availability as research bioresources. Cell lines were maintained in Dulbecco’s Modified Eagle Medium (DMEM) (Gibco-BRL Life Technologies, Auckland, New Zealand) containing 10% fetal bovine serum (FBS; Gibco-BRL Life Technologies, Auckland, New Zealand), 100 units/ml penicillin, and 100 μg/ml streptomycin at 37°C with 5% CO_2_. Cell lines were subcultured twice a week using a standard trypsinization protocol. Skin fibroblast cells (SF-A4) were a gift from Associate Professor Dr. Chanitra Thuwajit, Department of Immunology, Faculty of Medicine Siriraj Hospital, Mahidol University. 

*Preparation of peripheral blood mononuclear cells and human umbilical vein endothelial cells*

The protocols for the collection of peripheral blood mononuclear cells (PBMCs) and human umbilical vein endothelial cells (HUVECs) were approved by the Siriraj Institutional Review Board (SIRB) (COA No. Si405/2013). PBMCs from healthy donors were isolated by Ficoll-Hypaque Density Gradient Media (GE Healthcare, Freiburg, Germany). PBMCs were subsequently resuspended in Roswell Park Memorial Institute (RPMI) 1640 Medium (Gibco-BRL Life Technologies, Auckland, New Zealand). 

HUVECs were isolated from healthy term pregnancies. HUVECs were isolated from a 6-inch umbilical cord segment via trypsin digestion. HUVECs were cultured using M199 (Gibco-BRL Life Technologies, Auckland, New Zealand). 

*Cytotoxicity analysis*

Cytotoxicity was measured using PrestoBlue® Cell Viability Reagent (Invitrogen, Auckland, New Zealand). CCA cell lines and skin fibroblast were seeded in a 96-well microtitre plate at a concentration of 1x10^4^ cells/well, while PBMCs and HUVECs were seeded at 1x10^5^ cells/well of a 96-well plate for 24 h at 37°C and 5%CO_2_. The cells were exposed to the compound A (CpdA) [2-(4-acetoxyphenyl)-2-chloro-N-methylethylammonium-chloride], which was a gift from Dr. Irina Budunova, Department of Dermatology, Northwestern University, Chicago, Illinois, USA, with concentrations ranging between 1.56 µM and 250 µM. The microtitre plate was incubated for further 24 and 48 h and PrestoBlue^®^ Cell Viability Reagent was added. The plates were incubated for further 2 h where after the absorbance of the colour complex was read at 570 nm with a reference wavelength set at 600 nm for PrestoBlue^®^ Cell Viability Reagent, using a BIO-TEK multiwell plate reader.

*Cell cycle distribution analysis*

Cell cycle distributions were analyzed by measuring DNA contents that were stained with propidium iodide (PI) (Cayman Chemical, Ann Arbor, MI, USA). CCA cell lines were seeded in 6-well plates at a density of 1×10^5 ^cells/well in 2 ml of culture medium and incubated for 24 h. Cells were then treated with CpdA at indicated concentrations for 24 h. After CpdA treatment, cell pellets were collected, washed with cold phosphate-buffered saline (PBS), and fixed with ice-cold 70% ethanol. After the fixative was decanted, the cell pellets were resuspended in staining solution containing DNase-free RNase and PI. Cell cycle was determined using FACSortTM Flow Cytometer (Becton, Dickinson and Company, Franklin Lakes, NJ, USA), and the data was analyzed using CellQuestTM software (BD Biosciences, San Jose, CA, USA).

Real-time reverse transcription-polymerase chain reaction (RT-PCR)

To examine IL-6 transcription, total RNA was prepared from CCA cells untreated or treated with CpdA using Trizol™ Reagent (Invitrogen, Auckland, New Zealand), following the manufacturer’s recommended procedure. IL-6 mRNA was quantified by real-time RT-PCR technique using specific primers (forward primer sequence: 5’-GTACATCCTCGACG GCATC-3’ and reverse primer sequence: 5’-AGCCACTGGTTCTGTGCCT-3’). To examine TGFβR-II transcription, total RNA was prepared from CCA cells untreated or treated with CpdA and cisplatin as indicated by using Trizol™ Reagent (Invitrogen, Auckland, New Zealand), following the manufacturer’s recommended procedure. TGFβR-II mRNA was quantified by real-time RT-PCR technique using specific primers (forward primer sequence: 5’-ACGTTCAG AAGTCGGATGTGG-3’ and reverse primer sequence: 5’-TGCACTTTGGAGAAGCA GCA-3’). For both genes, cDNA synthesis was performed using Superscript®III Reverse Transcriptase (Invitrogen, Auckland, New Zealand), following the manufacturer’s recommended guidelines. Amplification of cDNA by real-time PCR was performed in a LightCycler^®^ 480 SYBR Green I Master (Roche LifeScience, Mannheim, Germany). The reactions were recorded and analyzed using a LightCycler^®^ 480 Instrument equipped with a 96-well thermal cycler (Roche LifeScience, Mannheim, Germany). Relative mRNA expression was normalized against GAPDH mRNA level (forward primer sequence: 5’-CGACCACTTTGTCAAGCTCA-3’ and reverse primer sequence: 5’-AGGGGTCTACAT GGCAACTG-3’) using a comparative Ct method (delta delta Ct).

*Cell proliferation assay*

The effect of CpdA and cisplatin on CCA cell proliferation was determined using PrestoBlue^®^ Cell Viability Reagent (Invitrogen, Auckland, New Zealand). CCA cells were seeded at 1×10^4^ cells/well in 96-well microtiter plates. After 24-h incubation, the cells were treated with indicated concentrations of CpdA and cisplatin (Sigma-Aldrich Corporation, St. Louis, MO, USA) for 24 h at 37^o^C in 5% CO_2_. Cell culture media was not refreshed during this period. At the end of incubation, PrestoBlueTM Cell Viability Reagent was added, after which the cells were incubated at 37°C until the reagent color changed. Metabolically active cells were stained. Absorbance was read at excitation of 570 nm and emission of 600 nm as a reference wavelength for normalization, and the percentage of cell survival was calculated. Data are reported as the percentage of cell survival compared to the percentage in CCA cells without treatment (set at 100%). 

*Statistical analysis*

All statistical analyses were performed using GraphPad Prism 6 Software (GraphPad Software, Inc., La Jolla, CA, USA). Mean and standard deviation (SD) from three independent experiments was calculated. The difference in experiment results was analyzed by one-way analysis of variance (ANOVA), followed by Tukey’s pos hoc test. A p-value less than 0.05 was considered statistically significant.

## Results

*Cytoxicity of compound A on primary cells*

**Figure 1:**
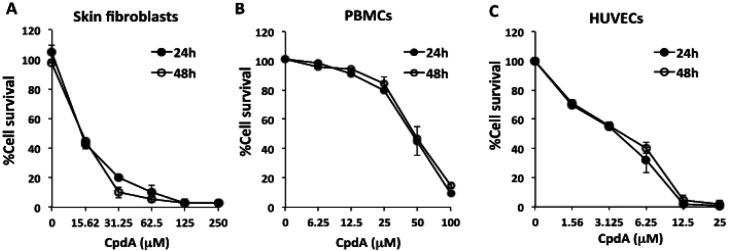
Effects of compound A (CpdA) on cytotoxicity in primary normal cells. Cells were treated with indicated concentrations of CpdA. After 24 h and 48 h, living cells were measured using PrestoBlue® Cell Viability Reagent, as described in the Materials and Methods section. (A) Skin fibroblasts, (B) PBMCs, and (C) HUVECs. Results are shown the percentage of cell survival compared to untreated control. The data represents the mean ± standard deviation from three independent experiments

To examine the cytotoxicity of compound A (CpdA) on primary normal cells, three types of primary cells, including skin fibroblast cells (SF-A4), peripheral blood mononuclear cells (PBMCs), and human umbilical vein endothelial cells (HUVECs), were tested. The effect of CpdA on cell survival was determined to observe the 50% cytotoxic concentration (CC50) of CpdA. After initial tests to determine appropriate concentrations, SF-A4 cells, PBMCs, and HUVECs were treated with CpdA at concentrations of 0-250 µM, 0-100 µM, and 0-25 µM, respectively, for 24 h and 48 h. The cell viability of each well was determined using PrestoBlueTM Cell Viability Reagent. The average cell survival obtained from triplicate determinations at each concentration was plotted as a dose-response curve. The CC50 of CpdA was defined as the lowest concentration that reduced cell growth by 50% in treated culture compared to untreated culture. At 24 h after treatment, the CC50 of CpdA for SF-A4, PBMCs, and HUVECs was 15.44±1.5, 49.86±3.33, and 3.22±0.61 µM, respectively. At 48 h after treatment, the CC50 of CpdA for SF-A4, PBMCs, and HUVECs was 13.23±3.38, 49.82±2.16, and 3.20±0.71 µM, respectively (Figure 1).

*Cytotoxicity of compound A on CCA cell lines*

**Figure 2:**
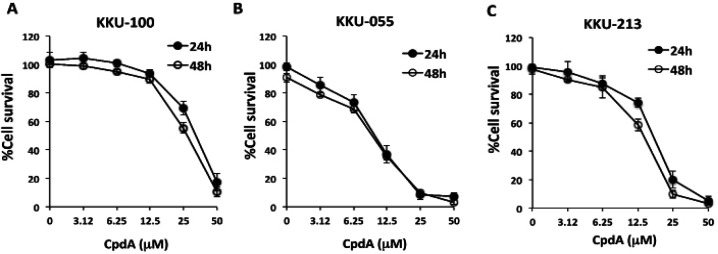
Effect of Compound A (CpdA) on Cytotoxicity in CCA Cell Lines. CCA cells were treated with indicated concentrations of CpdA. After 24 h and 48 h, living cells were measured using PrestoBlue® Cell Viability Reagent, as described in the Materials and Methods section. (A) KKU-100, (B) KKU-055, and (C) KKU-213. Results are shown the percentage of cell survival compared to untreated control. The data represents the mean ± standard deviation from three independent experiments

The cytotoxicity of CpdA on CCA cells was investigated in three CCA cell lines. KKU-100, KKU-055, and KKU-213 were treated with CpdA at concentration 0-50 µM for 24 h and 48 hr. PrestoBlueTM Cell Viability Reagent was then added, and the plate was incubated for an additional 2 h. The absorbance of the color complex was read at 570 nm with the reference wavelength set at 600 nm using a multiwell plate reader. As shown in Figure 2, the CC50 of CpdA at 24 h after treatment for KKU-100, KKU-055, and KKU-213 was 25.22±0.14, 12.28±0.34, and 12.99±0.31 µM, respectively. Moreover, at 48 h after treatment, the CC50 of CpdA for KKU-100, KKU-055, and KKU-213 was 24.84±1.12, 12.32±0.32, and 12.71±0.71 µM, respectively (Figure 2).

*Effect of compound A on cell cycle distribution in CCA cell lines*

**Figure 3:**
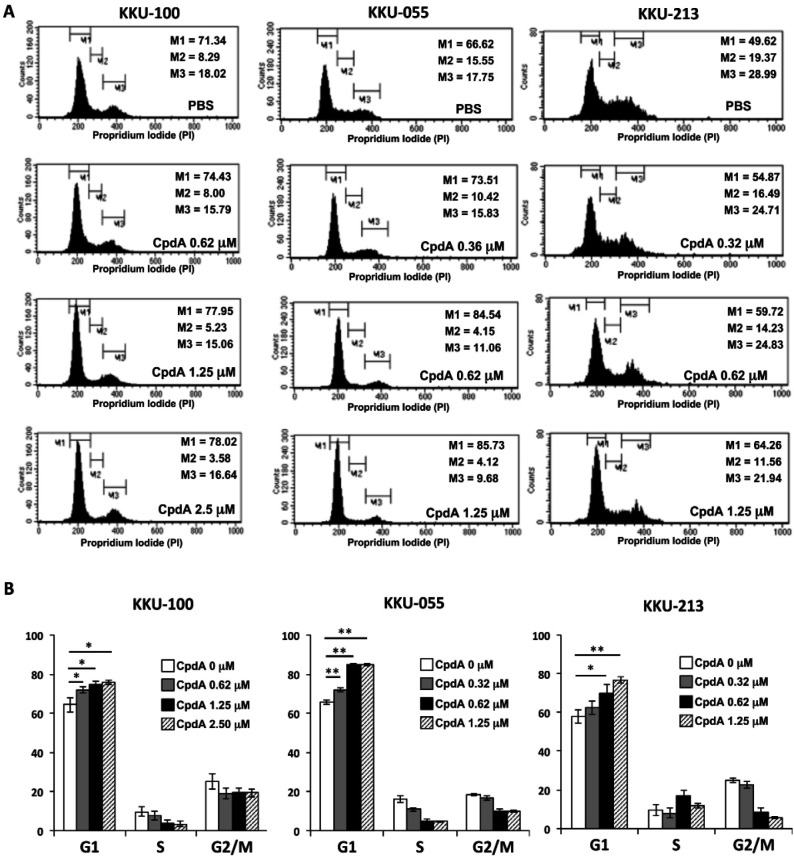
Effect of Compound A (CpdA) on Cell Cycle Distribution in CCA Cell Lines. KKU-100, KKU-M055, and KKU-M213 cells were treated with PBS or CpdA for 24 h. Cell cycle distribution was examined by propidium iodide (PI) staining, and then analyzed by flow cytometry. (A) Histogram of flow cytometry analysis. M1, M2, and M3 represent G0/G1, S, and G2/M phase, respectively. (B) The percentage of cells in each phase of cell cycle. The data represents the mean ± standard deviation from three independent experiments. Asterisks (*) indicate statistically significant difference when compared with parental expression (*p<0.05, **p<0.01)

The effect of CpdA on cell cycle distribution in CCA cell lines was investigated using propidium iodide (PI) staining. KKU-100 was treated with CpdA at 0.62, 1.25, and 2.5 µM, and KKU-055 and KKU213 were treated with CpdA at 0.32, 0.62, and 1.25 µM. After 24 h of incubation, treated cells were fixed and stained with PI. The G1, S, and G2-M phases of cell cycle were analyzed by flow cytometry. After CpdA treatment, the numbers of cells in G1 phase of KKU-100, KKU-055, and KKU213 cells were all significantly increased (Figure 3). These results suggest that CpdA inhibited CCA cell proliferation by inhibiting cell cycle at G1 phase.

*Effect of compound A on IL-6 expression in CCA cell lines*

**Figure 4:**
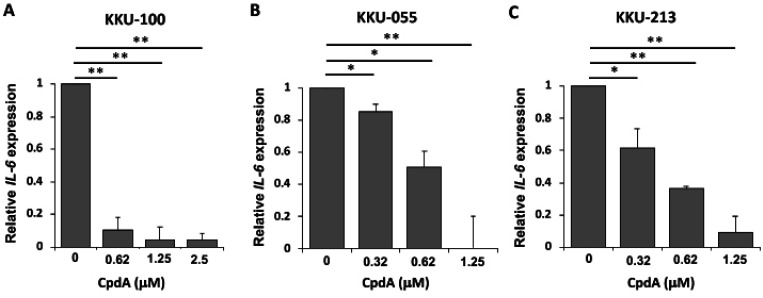
Effect of Compound A (CpdA) on IL-6 Expression in CCA Cell Lines. CCA cells were treated with the indicated concentrations of CpdA for 24 h. Relative gene expression (fold change) of IL-6 was determined by real-time PCR method in (A) KKU-100, (B) KKU-M055, and (C) KKU-M213 cells. The data represents the mean ± standard deviation from three independent experiments. Asterisks (*) indicate statistically significant difference when compared with parental expression (*p<0.05, **p<0.01)

To examine the effect of CpdA on IL-6 expression in CCA cell lines, sublethal doses of CpdA were used to treat the cells for 24 h. KKU-100 was treated with CpdA at 0.62, 1.25, and 2.5 µM, and KKU-055 and KKU213 were treated with CpdA at 0.32, 0.62, and 1.25 µM. After 24 h of incubation, IL-6 expression was determined by real-time PCR. As shown in Fig. 4A, IL-6 expression was markedly reduced in KKU-100 treated with 0.62 mM of CpdA. For KKU-055 and KKU-213, when treated with 0.32-1.25 µM of CpdA, IL-6 expression was decreased in a dose dependent manner (Figure 4B and 4C). These results indicate that CpdA reduced IL-6 expression in CCA cell lines.

*Effect of combination compound A and cisplatin treatment on CCA cell survival*

**Figure 5:**
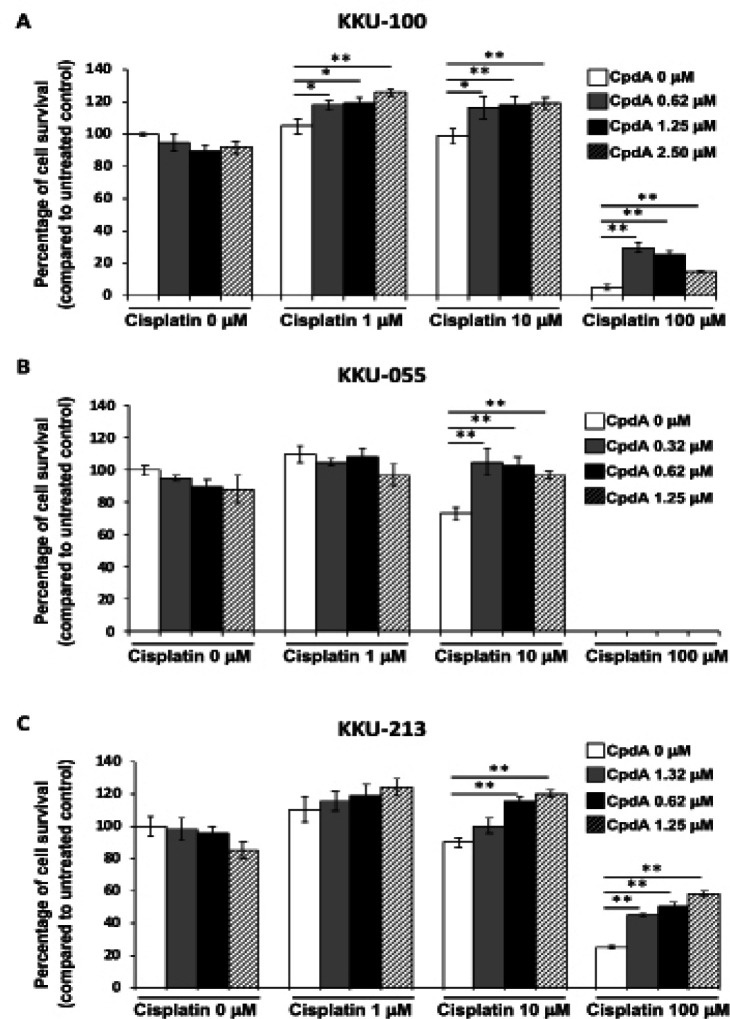
Effect of Combination Treatment with Compound A (CpdA) and Cisplatin on CCA Cell Survival. CCA cells were incubated with CpdA, or cisplatin, or their combinations at the indicated concentrations for 24 h. Cell survival (presented as percentage) of was determined in (A) KKU-100, (B) KKU-M055, and (C) KKU-M213 cells using PrestoBlue® Cell Viability Reagent, as described in the Materials and Methods section. The data represents the mean ± SD from three independent experiments (*p<0.05, **p<0.001)

IL-6 plays an important role in the pathogenesis and growth of CCA via the activation of several pathways involved in cell survival signaling. In previous experiments, it was observed that CpdA could reduce IL-6 expression and effectuate cell cycle arrest during G1 phase, and cisplatin is used for treatment of CCA. We were, therefore, inspired to investigate the combined effect of CpdA and cisplatin on CCA cell lines. Cell survival was determined using PrestoBlueTM Cell Viability Reagent. CpdA and cisplatin were combined and tested in the KKU-100, KKU-055, and KKU-213 CCA cell lines. CpdA at concentrations of 0.62, 1.25, and 2.5 µM for KKU-100, and at concentrations of 0.32, 0.62, and 1.25 µM for KKU-055 and KKU-213 were used in combination with cisplatin at 1, 10, and 100 µM. Combination CpdA and cisplatin treatments showed higher numbers of KKU-100 cell survival than the survival rates of cells treated with cisplatin alone (Figure 5A). The combinations of CpdA at 0.32, 0.62, and 1.25 µM with cisplatin at 10 µM also showed higher numbers of KKU-055 cell survival than the survival rates of cells treated with cisplatin alone (Figure 5B). For KKU-213, the combinations of CpdA at 0.32, 0.62, and 1.25 µM with cisplatin at 10 and 100 µM also showed higher numbers of cell survival than the survival rates of cells that were only treated with cisplatin (Figure 5C). These results indicate that combinations of sublethal doses of CpdA and cisplatin not only failed to inhibit CCA cell survival, this experimental combination therapy promoted increased CCA cell survival.

*Effect of compound A and cisplatin on TGFβReceptor-II expression*

**Figure 6:**
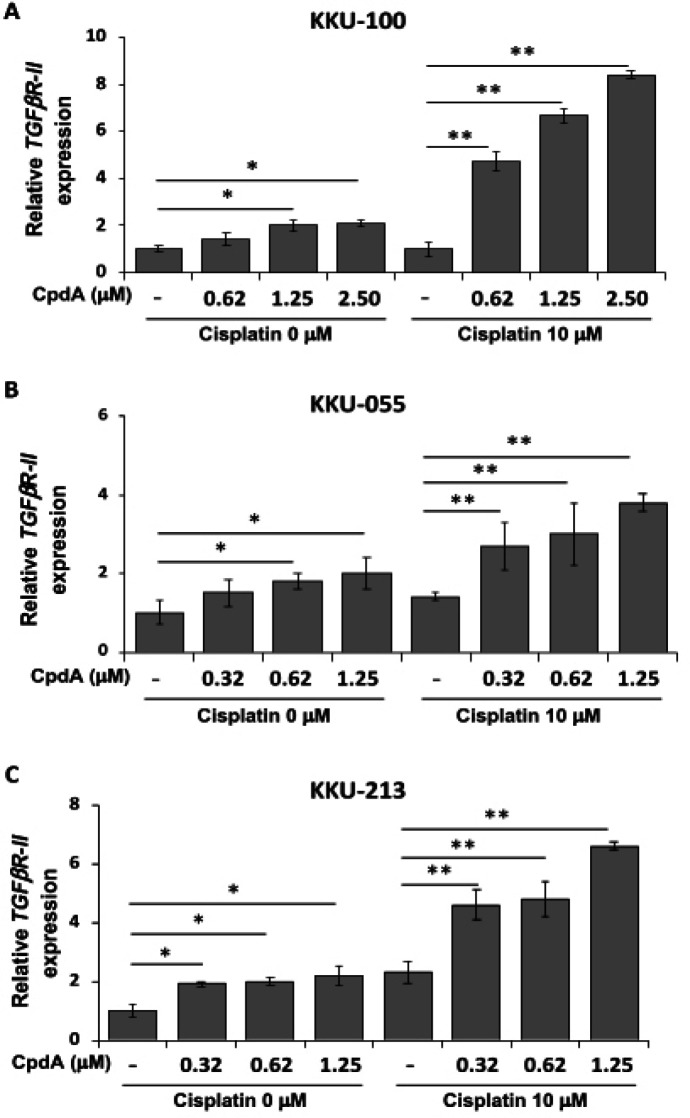
TGFβR-II Expression in CCA Cells Lines after Treatment with Combination Compound A (CpdA) and cisplatin. CCA cells were incubated with 10 μM of cisplatin, or CpdA at the indicated concentration, or their combinations for 24 h. Relative gene expression (fold change) of TGF*β*R-II was determined in (A) KKU-100, (B) KKU-M055, and (C) KKU-M213 cells by real-time PCR method. The data represents the mean ± standard deviation from three independent experiments. Asterisks (*) indicate statistically significant difference when compared with parental expression (*p<0.05, **p<0.01)

It was previously reported that glucocorticoid (GC) could upregulate TGFβ receptor II (TGFβR-II) expression, which induced cancer cell resistance to ciplatin (Lesovaya et al., 2011). Since CpdA has the same effect as GC, TGFβR-II expression was examined in the present study. Ten µM of cisplatin was used alone or in combination with CpdA at the indicated concentrations. TGFβR-II expression in treated CCA cells was determined by real-time PCR. As shown in Figure 6A, TGFβR-II expression was significantly increased (more than 4-folds; p<0.01) in combination cisplatin and CpdA-treated KKU-100 cells in a dose-dependent manner. Similar results were observed in combination cisplatin and CpdA-treated KKU-055 cells (more than 3-folds; p<0.01; Figure 6B) and KKU-213 cells (more than 4-folds; p<0.01; Figure 6C). These results indicate that a sublethal dose of CpdA induced TGFβR-II expression in CCA cell lines.

## Discussion

CCA is a lethal human cancer that evolves from a multistep carcinogenesis that involves chronic inflammation and alterations in cellular processes (Fava et al., 2007; Fava and Lorenzini, 2012). Proinflammatory cytokines, such as IL-6, play important roles in these altered cellular processes, in the onset of inflammation, and in the growth of CCA via activation of several survival signaling pathways (Okada et al., 1994; Park et al., 1999a; Park et al., 1999b). Dexamethasone, which is a synthetic glucocorticoid (GC), is a potent anti-inflammatory drug that is used for treatment of many chronic inflammatory diseases and cancers. GCs exhibit therapeutic effects by interacting with glucocorticoid receptor (GR) and cellular transcription factors (e.g., NF-λB and AP-1) (Robertson et al., 2010) that decrease proinflammatory, antiapoptotic, and survival gene expression, and these effects ultimately lead to tumor cell death (Chen et al., 2010). Dexamethasone was used to treat CCA, but several adverse metabolic side effects were observed (Pazdur et al., 1999). The side effects of GCs were found to be mediated by interaction of GR homodimer with responsive elements in promoters and enhancers of the genes implicated in metabolic controls (Halvorsen et al., 2003; Rao et al., 2011; Schlossmacher et al., 2011). In contrast to GCs, compound A (CpdA) is a small plant-derived GR modulator that harbors anti-inflammatory activities with fewer side effects (Louw et al., 1997; Louw and Swart, 1999; De Bosscher et al., 2005). CpdA strongly inhibits the growth and survival of prostate cancer and multiple myeloma cells by reducing proinflammatory cytokines (Yemelyanov et al., 2012; Lesovaya et al., 2013; Lesovaya et al., 2015). 

In the present study, the inhibitory effects of CpdA on CCA cell proliferation and IL-6 production were investigated. The combined effect of CpdA and the anticancer drug cisplatin was also evaluated. Initially, the cytotoxicity of CpdA was tested on primary normal cells, including skin fibroblasts (SF-A4), PBMCs, and HUVECs, and on the KKU-100, KKU-055, and KKU-213 CCA cell lines (Figure 1 and 2) to identify sublethal doses that are not toxic to the normal cells to be used for testing with CCA cell lines.

The effect of CpdA on cell proliferation was then investigated. The numbers of cells at the G1 phase of the cell cycle were significantly increased in CCA cell lines at 24 h after CpdA treatment (Figure 3). These results suggest that CpdA inhibited CCA cell proliferation at the G1 phase of the cell cycle. The antiproliferative effect of CpdA was previously reported in some other cancers. In leukemia cell lines (CEM and K562), CpdA inhibited cell growth and induced proapoptosis in these cells by activating trans-repression of NF-λB and AP-1 transcription factors (Lesovaya et al., 2011). The anticancer effect of CpdA was demonstrated via inhibition of both cell growth and the survival of highly malignant prostate cancer cells in a glucocorticoid receptor-dependent fashion (Robertson et al., 2010). CpdA strongly inhibited cell growth and viability in multiple myeloma cells and in human T-cell and B-cell lymphoma (Lesovaya et al., 2013). In combination with the proteasome inhibitor bortezomib, CpdA potently suppressed cell growth and survival by causing an accumulation of glucocorticoid receptors (Lesovaya et al., 2013).

CpdA has also been reported to inhibit cytokine expression and secretion in many inflammatory diseases (Reber et al., 2012). Several studies demonstrated that CpdA acts as anti-inflammatory agent by reducing the production of proinflammatory cytokines, such as IL-4, IL-5, and IL-13 (Reber et al., 2012). Regarding the molecular mechanism, CpdA binds to GR and induces its nuclear translocation (Rao et al., 2011). Subsequently, it suppresses the expression of proinflammatory cytokine genes encoding IL-6 and matrix metalloproteinase-1 (Dewint et al., 2008). Moreover, the anti-inflammatory effect of CpdA was reported to play a role in reducing DNA-binding activity and interferences with the transactivation potential of NF-λB (De Bosscher et al., 2005). A 2013 study reported that CpdA reduced TNFα-stimulated IκBα degradation and NF-kB p65 nuclear translocation in a heat shock factor 1-dependent manner in A549 lung epithelial cells. This effect diminished the expression of NF-kB-driven genes, such as IL-6 and IL-8 (Beck et al., 2013). IL-6 is one of the cytokines that increases and promotes CCA cell proliferation in CCA patients (Johnson et al., 2012). The effect of CpdA on IL-6 expression in the KKU-100, KKU-055, and KKU-213 CCA cell lines was examined and it was significantly decreased in CpdA-treated CCA cells (Figure 3), which suggests the inhibitory effect of CpdA on IL-6 expression in CCA cells. IL-6 plays an important role in the carcinogenesis and growth of CCA by activating several pathways involved in survival signaling (Johnson et al., 2012). Thus, the inhibitory effect of CpdA on IL-6 expression may at least partly explain its anticancer effect in CCA.

The anticancer drug resistance reported in CCA patients result in a need for increased doses of the drugs used. We hypothesized that CpdA combined with cisplatin might be a more effective treatment for CCA. The results of our experiments revealed no increased anticancer benefit from combination CpdA and cisplatin in CCA. Importantly and in contrast, the combination of CpdA and cisplatin had the effect of increasing CCA cell survival (Figure 5). This finding is supported by the results of a study of Chen (2010) that showed that combined treatment with dexamethasone and cisplatin increased the survival of ovarian cancer cell lines (Chen et al., 2010). It was previously reported that dexamethasone induced TGFβ receptor-II (TGFβR-II) expression and enhanced cell resistance to cisplatin via the TGF-β1 signaling pathway (Chen et al., 2010). Since CpdA exhibits action similar to that of dexamethasone, we hypothesized that the combination of CpdA and cisplatin increases CCA cell survival by induction of TGFβR-II expression. Thus, TGFβR-II expression was examined in the combined treatment of CCA cell lines with CpdA and cisplatin. The results of that investigation showed TGFβR-II expressions to be increased in a dose-dependent manner with cisplatin concentrations (Figure 6). Therefore, induction of TGFβR-II expression the promotes cell survival via the TGFβR-II signaling pathway may at least in part explain the resistance of CCA cells to the combined treatment. However, the effect of CpdA and cisplatin combination with either CpdA or cisplatin alone on IL-6 expression, cell cycle status, and other related molecules should be confirmed in further study.

The results of this study demonstrated that a sublethal dose of CpdA inhibited CCA cell cycle at G1 phase, and reduced IL-6 expression in CCA cell lines. However, instead of producing an increased anticancer effect, combination treatment with CpdA and cisplatin actually increased CCA cell survival. The molecular mechanism of the resistance of CCA cells to the combined treatment is likely attributable to increased TGFβR-II expression that promotes cell survival via the TGFβR-II signaling pathway. Combination CpdA and cisplatin is, therefore, not recommended for the treatment of CCA. The combination of CpdA with other anticancer drugs for CCA treatment should be further investigated.
